# Transcriptome and Metabolomic Analyses Reveal Regulatory Networks Controlling Maize Stomatal Development in Response to Blue Light

**DOI:** 10.3390/ijms22105393

**Published:** 2021-05-20

**Authors:** Tiedong Liu, Xiwen Zhang

**Affiliations:** 1 College of Agriculture, Fujian Agriculture and Forestry University, Fuzhou 350002, China; 2 College of Mechanical and Electronic Engineering, Fujian Agriculture and Forestry University, Fuzhou 350002, China; xwzhang@fafu.edu.cn

**Keywords:** stomata, transcriptome, metabolome, signaling transduction, blue light, maize

## Abstract

(1) Background: Blue light is important for the formation of maize stomata, but the signal network remains unclear. (2) Methods: We replaced red light with blue light in an experiment and provided a complementary regulatory network for the stomatal development of maize by using transcriptome and metabolomics analysis. (3) Results: Exposure to blue light led to 1296 differentially expressed genes and 419 differential metabolites. Transcriptome comparisons and correlation signaling network analysis detected 55 genes, and identified 6 genes that work in the regulation of the HY5 module and MAPK cascade, that interact with PTI1, COI1, MPK2, and MPK3, in response to the substitution of blue light in environmental adaptation and signaling transduction pathways. Metabolomics analysis showed that two genes involved in carotenoid biosynthesis and starch and sucrose metabolism participate in stomatal development. Their signaling sites located on the PHI1 and MPK2 sites of the MAPK cascade respond to blue light signaling. (4) Conclusions: Blue light remarkably changed the transcriptional signal transduction and metabolism of metabolites, and eight obtained genes worked in the HY5 module and MAPK cascade.

## 1. Introduction

The stomata is a specialized epidermal cell structure distributed on the leaf and stem surface of terrestrial plants. It is an important channel for the exchange of oxygen, carbon dioxide, water, and heat between plants and the environment. The morphology, distribution, movement, and development of the stomata directly determine plant physiological activities such as photosynthesis, respiration, and transpiration. Under changeable environmental conditions, plant sense outside changes of gas, water, heat, and light, and collaborate with endogenous factors by finely adjusting the stomatal number to optimize the exchange of gas, water, and heat to adapt to the environment. However, the unpredictability of environmental factors and the complexity of reacting internal signals bring great challenges to the research on the regulated signaling network of stomatal formation. The cell fate, structure formation, and cell cycle of the leaf stomatal lineage cells of monocotyledons, such as Gramineous maize, are quite different from those of dicotyledonous plants [1].

A large and complex regulatory signaling network controls the differentiation and distribution of stomata. The main function of this network is to divide the meristem and precursor cells on time to achieve the cell cycle of stomatal lineage cell. However, the influence of environmental factors on the stomatal formation regulatory network is difficult to explain because of the complexity of meristem development and differentiation. Light quality is one of the important factors that mediate stomatal development in plants and plays an important role in the different development mechanisms of monocotyledons and dicotyledons [2]. As an exogenous signal, blue light is the direct initiator of signal network regulation and a key environmental factor that determines the interaction signal at molecular levels [3,4]. External and internal factors must be considered to explain the regulation mode of blue light on the stomatal development in maize. The regulation process of blue light on stomatal development is closely related to the constitutively photomorphogenic 1 (COP1)–elongated hypocotyl_5 (HY5)–phytochrome interacting factor (PIF) pathway regulated by cryptochrome (CRY) [5]. Blue light affects the binding of the erecta (ER)–too many mouths (TMM) complex of the epidermal patterning factor (EPF) transcription factor in EPF1/2 negative regulation and STOMAGEN positive regulation in maize [6]; thus, expressed ER may regulate multiple developmental processes through perceiving different EPF, each with a specialized expression pattern [7]. As another signaling cascade, the mitogen-activated protein kinase (MAPK) module is the core of blue-mediated regulation in the stomatal development network. The MAPK module contains YDA, MKKs, and MPKs, which integrate internal and environmental signals [2]. Blue light participates in the direct signal transduction and indirect regulation of COP1–HY5–PIF and MAPKs and thus participates in regulation networks. The adjustment of blue light finally affects the adjustment of terminal molecular switches. The SPCH, MUTE, and FAMA proteins of the basic helix-loop-helix (bHLH) family act as molecular switches in every link of stomatal division. SPCH is expressed during the early stages of the asymmetric division of stomatal lineage and then enriched in meristems to promote the asymmetric division of stomatal lineage. Maize MUTE proteins may be expressed in the guard mother cell (GMC) first and enter the subsidiary mother cells (SMC) on both sides from the GMC, and then regulate the formation of two subsidiary cells (SCs). FAMA restricts GMC cell division and promotes guard cell (GC) transition. However, the expression of FAMA has no certain relationship with the formation of GC and SC [8]. Periodically, cell cycle starts the stomatal development process [9]. For maize, cell cycle promotes the increase of stomatal index after adapting to the signal of blue light [6,10]. Therefore, blue light plays a particularly important role that affects the stomatal development of maize.

At present, the research on stomatal development mediated by light still focuses on light intensity and red light [11]. The known pathway is not sufficient to understand the whole network of stomatal development in response to blue light. After all, the different modules regulated by environmental factors and endogenous metabolic modulators in the stomatal development form a complex regulatory network. Blue light provides energy for photosynthesis, and influences the growth and development of plants as an environmental signal. Consequently, plants are able to fully adapt and utilize the regulated information of blue light. However, the photomorphogenesis and photochemical processes of plants are species-specific for C4 plants in Gramineae. The mechanism of blue light signals should be further studied systematically. This project intended to reveal the intercellular signal transduction, the network and location of signaling cascades, and the regulated direction of metabolism in maize from the 2D analyses of the transcriptome and metabolome. The purpose of this experiment was to find and identify putative functional genes that affect the stomatal development of maize from the aspects of light-dependent signal transduction and key signal cascades.

## 2. Results

### 2.1. Gene Expression Profile in Light Treatments

The number and distribution of the gene expression value in samples have certain differences. Thus, the sample expression value (FPKM) was divided into different intervals, the number of genes expressed in the samples of different expression intervals was calculated, and the stacking histogram was drawn. The gene expression distribution map of the samples is shown in Figure S1a. The correlation analysis of all transcriptome samples shows a remarkable correlation between biological replicates (Figure S1a). The principal component analysis (PCA) of total unique genes shows that genes in each treatment were homogenously expressed at principal component 1 (PC 1) with 59.1% variance (Figure S1c). The clustering method was used to calculate the distance between samples to investigate the similarity between samples. Samples that belong to the same treatment are close in distance and preferentially clustered together. The cluster diagram of the sequencing samples obtained according to gene expression is shown in Figure S1d. The gene expression profiles indicate that blue light brings a large number of differentially expressed genes (DEGs).

### 2.2. DEGs in Maize That Respond to Blue Light

**Figure 1:**
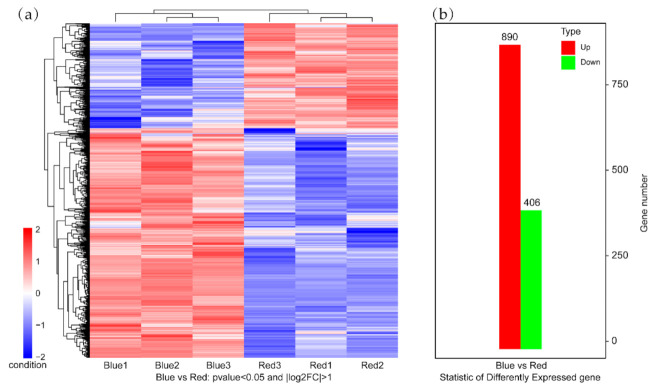
Cluster analysis results of different groups (**a**). Statistic of different expressed genes (**b**), in which the red plots show the number of significantly up-regulated genes and green plots show the number of significantly down-regulated genes.

The results of the transcriptome analysis of the maize leaf samples are listed in Table S1. More than 8 G of data was obtained in the RNA-seq libraries with GC > 56.23% and Q30 > 93.92%. Approximately 92.85% (blue) and 92.97% (red) clean reads were mapped to the reference genome of maize. In total, about 89.25% (blue) and 88.97% (red) of the unique genes were uniquely mapped to the reference genome. A total 1296 genes were differentially expressed. Among them, 890 were up-regulated and 406 were down-regulated when the light was changed from red to blue (Figure 1b). The DEGs were subjected to unsupervised hierarchical clustering. Genes in the same cluster may have similar biological functions for further analysis (Figure 1a).

### 2.3. Gene Ontology (GO) and Kyoto Encyclopedia of Genes and Genomes (KEGG) Analyses of the DEGs

**Figure 2:**
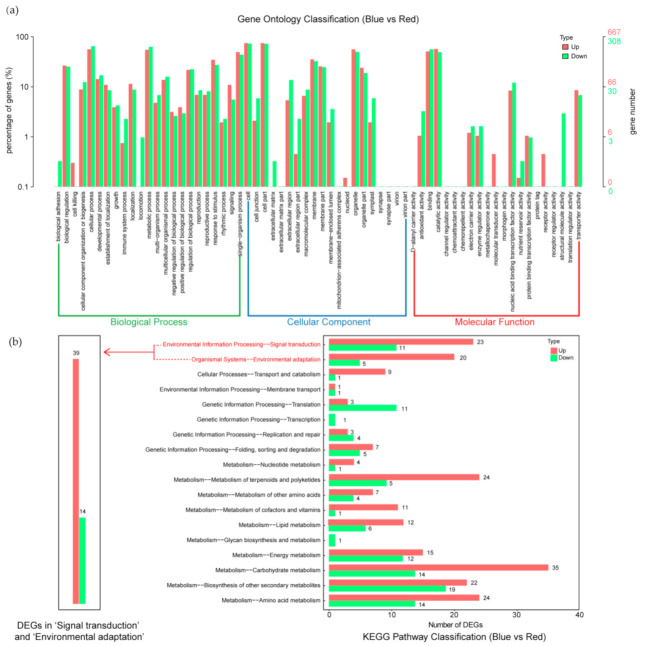
DEG gene ontology classification (**a**) and KEGG pathway classification (**b**) of maize transcriptome after light change from red to blue.

GO analysis was carried out to analyze the functions of the DEGs that respond to the change in light condition. The summary of GO categories is depicted in Figure 2a. According to the GO classification, DEGs involved in biological process occurred after light condition change. A total 308 genes were down-regulated in level 2 GO analysis; 198, 193, and 135 genes were involved in the top three GO terms under biological process, namely, “cellular process”, “metabolic process”, and “single-organism process”, respectively; 224, 223, and 154 genes were involved in the top three GO terms under cellular component, namely, “cell”, “cell part”, and “organelle”, respectively; and 173, 151, and 37 genes were involved in the top three GO terms under molecular function, namely, “binding”, “catalytic activity”, and “nucleic acid binding transcription factor activity”, respectively. In total, 667 genes were up-regulated in GO analysis. For biological process, 375, 367, and 328 genes involved in “cellular process”, “metabolic process”, and “single-organism process” were up-regulated, respectively. For cellular component, 500, 500, and 375 genes related to “cell”, “cell part”, and “organelle” were up regulated, respectively. For molecular function, 339, 381, and 57 genes related to “binding”, “catalytic activity”, and “transporter activity” were enriched, respectively. The results of the GO analysis were used for the functional analysis of the screened genes (Figure 2a).

KEGG analysis showed that the DEGs were remarkably enriched in “plant hormone signal transduction”, “MAPK signaling pathway”, “glycerophospholipid metabolism”, “starch and sucrose metabolism”, and “circadian rhythm” (Figure 2b). The “signal transduction” and “environment adaptation” processes of maize leaf stomata in response to blue light were collected. According to the KEGG pathway identification, 53 genes were classified into four major enrichment classes, namely, “plant hormone signal transduction”, “MAPK signaling pathway”, “plant pathogen–interaction”, and “circadian rhythm–plant”, and six less enriched classes, namely “inositol phosphate metabolism”, “phosphatidylinositol signaling system”, “endocytosis”, “ubiquitin mediated proteolysis”, “amino sugar and nucleotide sugar metabolism”, and “protein processing in endoplasmic reticulum”. The enrichment terms and annotations of these 53 DEGs are listed in Table S2. Gene annotations were retrieved from National Center for Biotechnology Information (NCBI) and InterPro. Four DEGs (LOC100281912, LOC107326007, LOC107403167, and LOC100192073, accessed on 20 April 2021) were not fully annotated in the KEGG analysis.

### 2.4. Protein–Protein Interaction (PPI) Network Analysis of Correlated DEGs and Identification of Potential Functional DEGs

**Figure 3:**
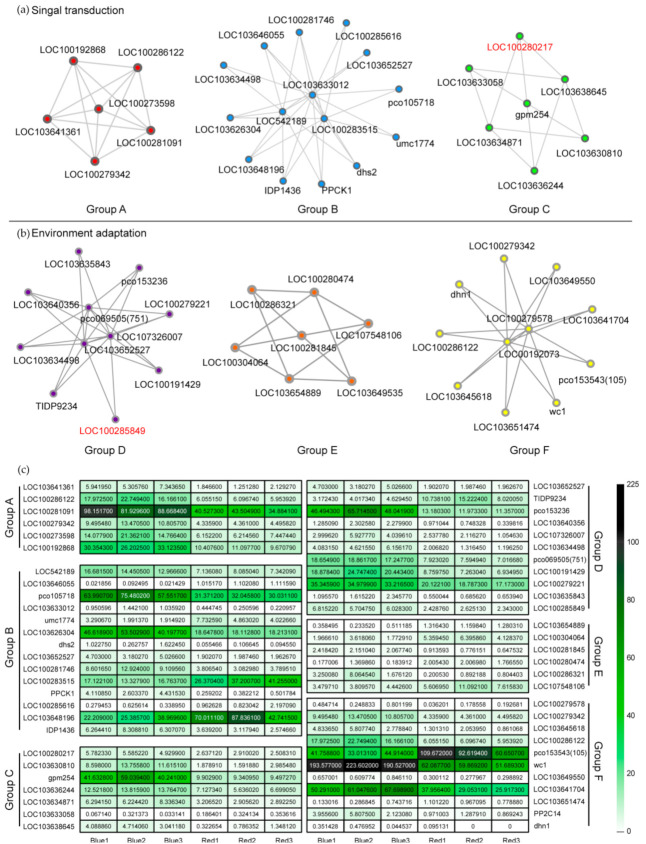
Six nods obtained from MCODE analysis of DEGs, 3 of ‘signal transduction’ (**a**) and 3 of ‘environment adaptation’ (**b**). The heat map (**c**) represents the transcription expression levels of 55 genes with gene interaction, which were divided into two large groups and six subgroups.

The correlation network of 53 DEGs in “signal transduction” and “environment adaptation” (according to KEGG classification level 2) were conducted in all transcriptome samples to fully understand the expression of the genes responding to light change in maize seedling stage (Table S2). The PPI network of the DEGs was constructed and classified by MCODE according to the correlation of DEGs (Figure 3a,b). Three nodes and 25 genes were obtained under the KEGG pathway, “environment adaptation” (Figure 3a). There were 27 genes within three nodes obtained in the “signal transduction” KEGG pathway (Figure 3b). The differences in the transcription expression of these 55 genes are shown in the heat map (Figure 3c). Three repetitive genes were deleted, and 24 genes unrelated to the KEGG pathway, but that interacted with the 28 annotated genes in Table S3, were finally obtained Twenty-two of the 24 genes can be annotated from NCBI and GO (Table S3). The other two genes, namely, LOC100280217 and LOC100285849, were unidentified.

**Figure 4:**
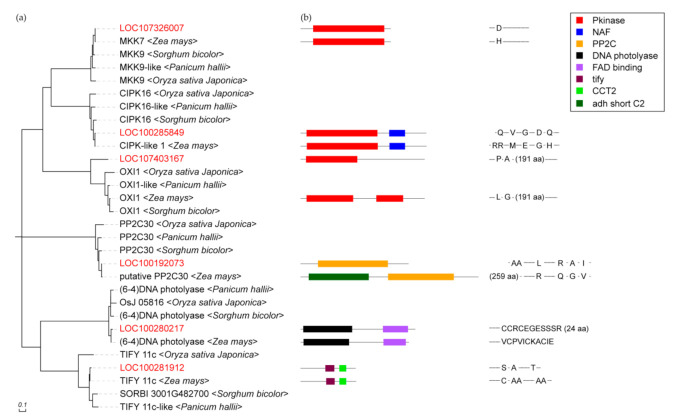
Part (**a**) represents the BLASTP results of 6 incompletely identified genes obtained by comparing with maize protein sequence and other three Gramineae plants. Part (**b**) shows the protein domain and sequence difference retrieved from BLASTP of 6 unidentified genes.

Six unidentified genes obtained from the KEGG and PPI of maize and three Gramineae plants, namely, *Oryza sativa japonica*, *Panicum hallii*, and *Sorghum bicolor*, were analyzed using the Protein Basic Local Alignment Search Tool (BLASTP) in the NCBI protein database (Figure 4). The BLASTP results showed that the six genes belong to different gene families. LOC107326007 is similar to MKK7 (Per. Ident = 99.39%) and MKK9 (Per. Ident = 91.44%) in the MAPK family. MKK7/9 are key members of the MAPK cascade that controls the connection pathway between ligands to receptors in the stomatal development signal transduction. LOC107326007 best matched with MKK7 than MKK9 in maize. Therefore, homologous BLASTP to other Gramineae plants suggest that LOC107326007 may be the MKK7 of maize (Figure 4a). LOC100285849 is identified as the CIPK16 (98.7 with CIPK-like 1) of the CBL-interacting protein kinase family. Its signaling pathway involved in stomatal development probably started with the catalysis of gamma-phosphoryl on protein substrates and/or calcium signals triggered by light. LOC107403167 is deduced to be a member of serine/threonine protein kinases. The protein sequence is closed, but the matching degree with the OXI1 of maize is low (query cover = 58%); thus, its gene function cannot be determined from existing results. LOC100192073 is determined as the PP2C14 of the protein phosphatase 2C (PP2C) family. The BLASTP results reveal that LOC100192073 and PP2C30 are close, but an additional NADB_Rossmann protein domain is found in putative maize PP2C30 (Figure 4b). Therefore, LOC100192073 is not PP2C30 but a member of the PP2C family. LOC100280217 with conserved PhrB domain interacts with HY5 and is identified as (6–4) DNA photolyase (query cover = 92%), similar to UVR3_1, but it is not reported in maize. Therefore, the effect of (6–4) DNA photolyase on the signal pathway of stomatal development in maize is not clear. LOC100281912 is close to the TIFY 11c (Per. Ident = 98.04%) of maize and other Gramineae plants, but has two differences and more than two amino acids in length. Noticeably, the BLASTP results indicate that LOC100281912 is also close to the pnFL-2 (Per. Ident = 98.03%) of maize.

### 2.5. Metabolite Profile and Screening of Differential Metabolites in Maize in Response to Light Change

**Figure 5:**
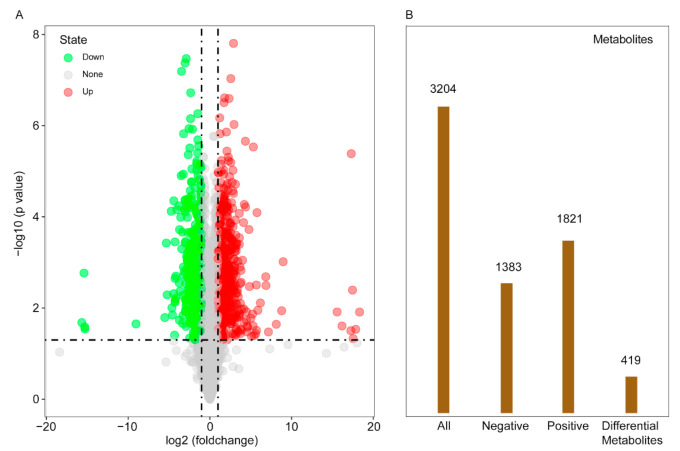
Volcanic diagram (**A**) represents all peaks of the total of 6272 detected metabolites. Where the red dots represent the significantly up-regulated, the green dots represent the significantly down-regulated, and the gray points represent the insignificant differential metabolites. Histogram (**B**) represents the scanning results from negative scanning mode and positive scanning mode, and final differential metabolite quantity filtered by the OPLS-DA method.

A total of 6272 peaks were detected by liquid chromatography–tandem mass spectrometry analysis, and 3204 metabolites were annotated in the MS2 database. Among them, 1383 metabolites were obtained by negative ion scanning modes and 1821 metabolites were obtained by positive ion scanning modes (Figure 5B). All metabolites were analyzed by principal component analysis and hierarchical cluster analysis to determine the influence of blue light on the change of metabolites (Figure S2). A clear separation was found between the two sampling responses to light change. A volcanic diagram was used to screen different metabolites by visualizing the *p* value and fold change value from the 6272 peaks (Figure 5A). Multi-dimensional analysis and one-dimensional analysis were used to screen different metabolites between groups after the Student’s test and fold change analysis of the 3204 detected metabolites. As a result, 419 different metabolites were obtained by orthogonal partial least squares discrimination analysis (OPLS-DA) (Figure 5B).

**Figure 6:**
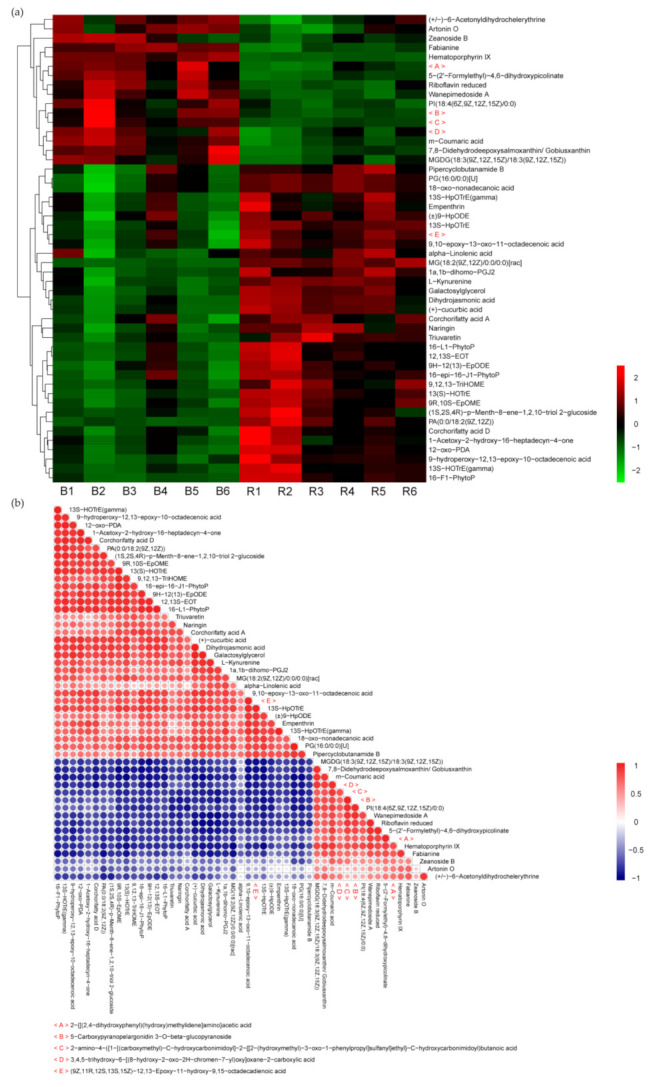
The heat map represents the transcription expression levels of 55 genes with gene interaction, which were divided into two large groups and six subgroups. Hierarchical clustering map (**a**) of the expression of the top 50 different metabolites. The x-coordinate represents the sample name, and the y-coordinate represents the differential metabolite. From green to red, the abundance of metabolites is from low to high. Correlation analysis chart of TOP-50 differential metabolites (**b**). The color from blue to red indicates the negative to positive correlation of metabolites.

Hierarchical clustering and visual analysis were conducted on the expression levels of the top 50 differential metabolites according to the variable importance of projection (VIP) values to show the relationship and the expression differences of metabolites between samples (Figure 6a). Correlation analysis was helpful to measure the degree of correlation and the relationship between significantly different metabolites. Pearson correlation coefficient was used for correlation analysis, and Pearson product difference correlation coefficient was used to measure the linear correlation between two quantitative variables (Figure 6b). The differential metabolite expression amount was visualized according to the VIP value. Figure 6b shows the correlation of the top 50 differential metabolites.

### 2.6. Effect of Light Change on Metabolic Pathways and Gene Screening in the Joint Analysis of the Transcriptome and Metabolome

**Figure 7:**
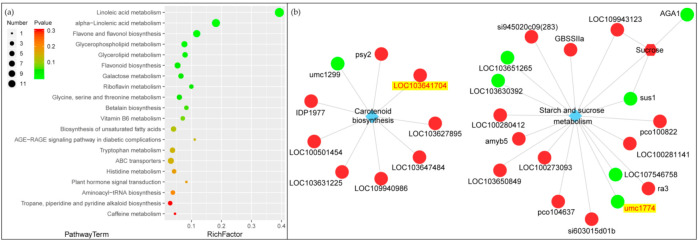
Enrichment analysis of metabolic pathways of different metabolites based on KEGG database (**a**). *p*-value in the metabolic pathway is the significance of enrichment of this metabolic pathway, and the significant enrichment pathway is selected to draw the bubble chart. The color from red to green indicate a continuous decrease in *p*-value. The scale of dots represents the number of metabolites. In the KGML network diagram of genes and metabolites (**b**). The circle represents the protein, the hexagon represents the metabolite, and the diamond represents the pathway name. Red indicates up-regulated proteins or metabolites, while green indicates down-regulated proteins or metabolites.

The function and interaction of metabolites were established by using the KEGG database. The enrichment analysis of differential metabolites by analyzing metabolite-related metabolic pathways is helpful to understand the mechanism of metabolic pathway changes in different samples. The analysis of the differential accumulation of metabolites in stomatal development showed that 65 metabolites accumulated differentially after 12 h of blue light change. Among the metabolites, 31 were up regulated and 34 were down regulated. The key metabolites affecting stomatal development during the light change from red to blue, such as those under the “carotenoid biosynthesis” and “starch and sucrose metabolism” pathways, were analyzed (Figure 7a).

The KEGG Markup Language (KGML) file in the KEGG database was used to map the network of genes and metabolites. This map will contribute to a more systematic study of the interactions between transcriptome and metabolomics. The KGML results showed two genes, LOC103641704 and umc1774, identified in “carotenoid biosynthesis” and “starch and sucrose metabolism” of the KEGG pathways in the joint analysis that affected the genes related to environmental adaptation and signal transduction (Figure 7b). LOC103641704 is a zeaxanthin epoxidase of the chloroplastic. Umc1774 is an alpha-1,4-glucan phosphorylase called starch phosphorylase1.

### 2.7. Location of Key Genes and Their Expression in the Network of Stomatal Development

**Figure 8:**
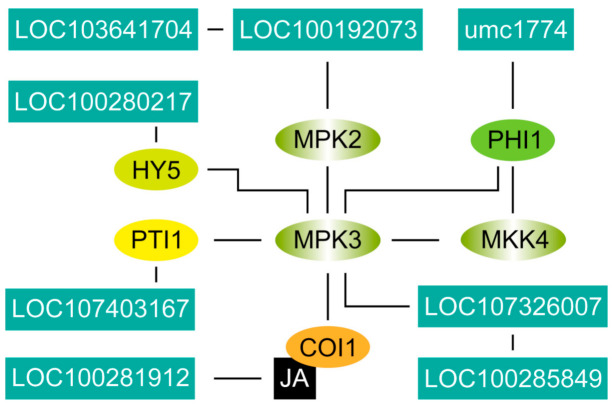
The probable location of 6 putative genes of maize in the network which is constructed with STRING database. The position of LOC100192073 searched from *Oryza sativa*. LOC100281912 is retrieved from *Panicum virgatum*.

Four (LOC107326007, LOC107403167, LOC100192073, LOC100281912), two (LOC100285849, LOC100280217), and two (LOC103641704, umc1774) genes were obtained from the KEGG pathway analysis, PPI, and KGML (Figure 8). The LOC107326007 of putative MKK7 is assumed to be located in the MAPK cascade between YDA and MPK3. LOC100285849 is close to LOC107326007 because of the interactions. LOC100280217 is located next to HY5 with the existing PPI. LOC107403167 is identified as a member of the OXI1 family from the MAPK pathway, and the interaction among LOC107403167, MKK4, and MPK3 were retrieved from the Search Tool for the Retrieval of Interacting Genes/Proteins (STRING) database by PPI through the connection with PTI1. Considering the exclusion of additional domains, the PP2C30 protein of *Oryza sativa* was used for protein interaction selection. Lastly, a protein signaling pathway from PP2C30 to MPK3 was established. Therefore, we speculated that LOC100192073 is also located in the signal network near MPK3. TIFY 11c is involved in jasmonate signaling. Thus, we inferred that LOC100281912 interacts more strongly with MPK3 by COI1 [12]. The association between LOC100281912 and MPK3 was retrieved from STRING through the reported protein of Panicum virgatum. LOC103641704 interacts with the PP2C of LOC100192073, and umc1774 connects MKK4 and MPK3 by linking with PHI1. The results showed that the potential genes work on the regulatory nodes of HY5 and MPK3.

**Figure 9:**
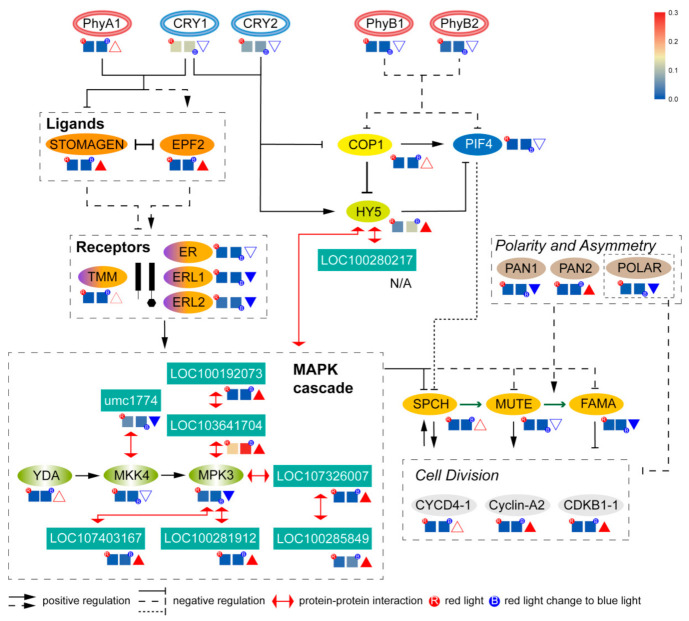
Basic protein signaling network of stomatal development and the qPCR expression of genes corresponds to the protein in the network. The experimental confirmation steps are indicated by dashed lines, and the uncertain steps are indicated by broken lines. Squares represent the expression quantity, triangles represent the up-regulated expression, solid triangles represent the significantly up-regulated expression, inverted triangles represent the significantly down-regulated expression, and solid inverted triangles represent the significantly down-regulated expression.

The basic network signal map was drawn according to the reported stomatal signaling pathway of monocotyledons (Figure 9). The expression of 32 genes, including 26 genes that play important roles in regulating the stomatal development of maize and 6 potential functional genes obtained in this experiment, were determined by qPCR (Figure 9). The qPCR results were consistent with the results of transcription expression. The 26 genes were divided into eight modules according to function location. The expression of photoreceptor genes *CRY1/2*, *phytochrome (Phy) A1*, and *PhyB1*/*2* did not change significantly. The two genes in the “Ligands” module, namely, *STOMAGEN* and *EPF2*, were remarkably up-regulated. *ERL1*/*2* in the “Receptor” module were significantly down-regulated. *MPK3* was significantly down-regulated in “MAPK cascades”. Only *HY5* showed significant up-regulation changes in the “COP1–PIF4–HY5” module in the core of the network. Among the three genes on the molecular switch, only *FAMA* was decreased significantly. The expression of *POLAR* and *PAN1* in the “Polarity and Asymmetry” modules were significantly lower, whereas the expression of *PAN2* was remarkably higher. The expression levels of *Cyclin-A2* and *CDKB1-1* in the “Cell Division” module was significantly up-regulated. The expressions of other genes were not remarkably as shown in Figure 9. The quantitative expression of the eight potential genes and their protein network locations are shown in the Figure 9. Among them, the expression of six genes increased, the expression of one gene decreased, and the expression of one gene was too low to be detected.

## 3. Discussion

### 3.1. Effects of Blue Light on Transcription Expression and Metabolite Formation in Maize Leaves

Light change has a remarkable effect on the transcription expression of maize seedling leaves. A large number of differential genes are affected by light change. The inadequate annotation of the maize genome and the number of unidentified proteins brought new challenges to the research on the response of maize stomatal development network to environmental change. The network can be mapped and the protein function at a specific location can be preliminarily inferred based on other reported Gramineae plants. In this study, 1296 DEGs and 419 differential metabolites were found. Among these DEGs, 53 genes directly influence the adaptation of plants to the environment and the subsequent signal transduction. Two genes, related metabolites, and their metabolic networks were obtained by analyzing the differential metabolites of the differential genes. The differential genes indicated that maize reacted in the signal pathway after red light was converted to blue light; therefore, the variation of downstream metabolites was determined. The stomatal development of plants involves complex network signals, including the regulation of transcription, protein, and metabolic levels [13,14,15]. Our results also showed that, at the transcription level, light signal is the main initial factor that influences downstream regulation in response to an environmental signal. At the metabolic level, the metabolic pathways of “carotenoid biosynthesis” and “starch and sucrose metabolism” may also participate in stomatal development.

### 3.2. Complement Network of Stomatal Development in Maize by PPI and KGML

PPI plays an important role in the transcription and signal transmission between cells. Determining the interactions between proteins can provide information about the functional structure and relationship of cells [16]. Key network genes (26 genes) and the genes (27 genes) obtained from the KEGG signaling pathways, “environmental adaptation” and “signal transduction”, were used as templates to search for PPI. Environmental light mediates the signal connection between activator and deactivator. There are 55 differential proteins that interact to form six clusters with their respective nodes (Figure 3). Twelve out of the 28 genes unannotated in the KEGG pathway represent proteases, such as MAPK [17], serine/threonine phosphatase [18], deoxyhypusine synthase [19], and 4-coumarate--CoA ligase [20], which can change the signaling response to light. The key node starts from the photoreceptor of stomatal development signal and is connected with the downstream of Suppressor of phyA-105 (SPA) as a partner of COP1 [21]. Calcium signal regulates the signaling activity of the protease, which, in turn, affects the cell cycle [22]. In addition, SLAH is expressed in guard cells [23]. Six uncharacterized genes that interact with HY5 and the MAPK cascade were obtained after clustering. These results indicate that these 28 genes are directly or indirectly involved in cell differentiation or stomatal separation induced by light regulation.

Two annotated genes were obtained by KGML analysis, which links the metabolites with the corresponding transcription level changes. In most plants, light promotes carotenoid biosynthesis and storage. Carotenoid accumulation is positively correlated with photomorphogenic development [24]. Cryptochromes (CRYs) transduce blue light signal by regulating the stability of transcription factor HY5. HY5 is a direct activator of the PSY gene, which accumulates carotenoids [25]. Meanwhile, strigolactone shares biosynthetic precursors with abscisic acid (ABA), both of which are derived from carotenoids [26,27]. They can interact with COP1–HY5 and affect stomatal development [28]. The MAPK signaling pathway is closely related to glucose [29,30]. Therefore, as precursors, the synthesis and transportation of starch and sucrose affect the MAPK pathway, and subsequently affect the stomatal development.

### 3.3. Regulation of the Key Nodes of HY5 and MAPKs by Blue Light Was the Determining Factor for the Differences in Stomatal Development

Transcription factor HY5 is a hierarchical regulator of transcription cascades in the photomorphogenesis that acts on the downstream aspect of many photoreceptors and serves as a signal bridge between light and metabolite [31]. First, HY5, as a target of the COP1–SPA complex, is regulated by light and metabolites at the protein and transcription levels [32,33,34]. Second, HY5 bridges light and metabolites signals and promotes light morphogenesis at the transcription level by activating the gene transcription of metabolite signaling pathways [35]. The reported work shows that photolyase genes are controlled by the transcription of HY5/HYH and induced by light exposure [36]. The (6–4) photolyases reverse the formation of photodimer and use blue light energy to restore the original base and avoid DNA damage [37]. Therefore, the increased expression of *LOC100280217* may be related to blue light induction and increased *HY5* expression. The detection of DNA damage is an important process to resist and tolerate environmental factors that cause damage. DNA photolyases take part in the network of proteins that is employed to recognize damage and initiate a signaling cascade that inhibits the progression of the cell cycle [38]. Although the expression level of (6–4) DNA photolyase was increased under blue light, it did not form cell cycle arrest by inhibiting CDKB1-1 [38]. This result indicates that HY5 may play a dominant role in the signal pathway of the HY5–(6–4) DNA photolyase cascade.

MPK3 in the MAPK cascade is a key signal regulator that controls organ development and responds to abiotic stress [39,40]. MPK3 is the center of ligand signal transmission to molecular switches in the stomatal development regulated by blue light. Several potential functional genes identified in this study are associated with MPK3 at PPI levels. PP2C interacts with ABA receptors and downstream SnRK2s. MPK2, which is the substrate of SnRK2s, is activated by MKK3 simultaneously in response to ABA [41,42]. Therefore, LOC100192073 is involved in the ABA signaling pathway and the MAPK cascade reaction at the same time. *LOC107326007* was identified as the homologous gene of *MKK7*, which is the key node in the MAPK cascade that controls stomatal development and transmits upstream signals to MPK3/4 [43]. The CBL-CIPKs of LOC100285849 form a signal cascade with MKK7/9 as induced by Ca^2+^ [44]. Therefore, we inferred that LOC100285849 competes with MKKs on calcium channels and participates in stomatal development. LOC107403167 was identified as a member of the STPK family, but it only has one Pkinase domain, whereas OXI1 has 2 Pkinase domains, as determined through BLASTP. The results showed that the function may be similar, but the differential domains suggest that they are not the same protein. However, interactions with MPK3 and known signal relationships between the STPK family and cyclins indicate that this gene may be deeply involved in stomatal development [45]. OXI1 affects cyclins and cyclin-dependent kinases (CDKs) by the H2O2 pathway [46]. LOC100281912 has a tify domain and a CCT2 domain, and its protein sequence is similar to that of TIFY 11c of the TIFY JAZ subfamily. Therefore, it may be a homologous allele of the TIFY family of maize. JAZ of the TIFY transcription factor subfamily protein may act as a repressor of jasmonate responses and mediate the interaction between JAZ proteins, a transcription repressor, and COI1 protein, a receptor [47]. Ca^2+^, MAPKs, and other factors constituted the JA signal cascade [48]. This composition may be the reason why LOC100281912 interacts with MPK3 protein and participates in stomatal development.

LOC103641704 and umc1774 were characterized in the maize genome sequence. 103641704 is a zeaxanthin epoxidase and linked to 100192073 through interaction with SnRK1. umc1774, which co-occurs with MKK4, MPK3, and PHI1 in genomes, is an alpha-1,4 glucan phosphorylase and an important allosteric enzyme in carbohydrate metabolism. Transcription and metabolism analyses showed that these two genes influence stomatal development through their respective signal networks and form bridges between protein signals and sugar networks.

### 3.4. Speculation of the Signaling Pathway of Blue Light Affecting the Stomatal Development of Maize

Key nodes in the known stomatal development network of monocotyledon plants were selected to construct the signal transmission network. These nodule genes and DEGs obtained from a transcriptome analysis were used for PPI relationship construction and clustering, but no DEGs were obtained for relationship mapping, except *HY5*. Therefore, the signal network in maize transcriptome concentrated on MAPKs and related signal cascades when light was changed to blue. The depicted signal networks show an interpretable signal process. The increase in ligand protein activity will promote ligand–receptor pair binding in the next step [49]. There were no substantial changes in the level of YDA, as the head of the MAPK cascade; therefore, the competitive binding between ligands and receptors may still be conservative. The remarkable increase in *HY5* expression in the COP1–HY5–PIF4 pathway may be the reason for the significant decrease in MPK3 expression at the end of the MAPK cascade [2]. Existing research on monocotyledons could not prove the regulatory relationship between MPK3 and the molecular switch FAMA. However, FAMA negatively regulates cyclins and CDKs [7]; blue light promotes the increase in the stomatal density and stomatal index of maize possibly through this mechanism [6,10].

## 4. Materials and Methods

### 4.1. Plant Material and Growth Conditions

Maize seed Xianyu 335 is a production of DuPont Company (Wilmington, DE, USA). The maternal part is PH6WC and the paternal part is PH4CV. Maize seedlings were cultivated in a climate-controlled cultivation chamber, designed and manufactured by the Center of Excellence for Research in Optoelectronic Agriculture at Fujian Agriculture and Forestry University. Uniform blue and red Light-emitting diode (LED) lamps were distributed on the top of each chamber. Maize seeds were cultivated under two light treatments: 660 nm red alone (Red) and 660 nm red, then 450 nm blue (Blue). The germinated maize seeds were transferred into blue light for 12 h, before the differentiation of fourth leaf under red light. Light density was set at 150 μmol m^−2^ s^−1^. Forty-eight seedlings were cultivated in a light chamber for a 24-h photoperiod every day, and each treatment was repeated four times. The temperature and relative humidity of the cultivation environment were set at 25 °C and 70%, respectively. Each fourth leaf bud was collected for subsequent experiments when it began to differentiate.

### 4.2. RNA Sequencing and Transcriptome Analysis

Total RNA was extracted using the mirVana miRNA Isolation Kit (Ambion) following the manufacturer’s protocol. RNA integrity was evaluated using the Agilent 2100 Bioanalyzer (Agilent Technologies, Santa Clara, CA, USA). The samples with RNA Integrity Number (RIN) ≥ 7 were subjected to the subsequent analysis. The libraries were constructed using TruSeq Stranded mRNA LTSample Prep Kit (Illumina, San Diego, CA, USA) according to the manufacturer’s instructions. Then, these libraries were sequenced on the Illumina sequencing platform HiSeqTM 2500 and 125bp/150bp paired-end reads were generated. Raw reads of sequencing were processed into clean reads by filtering low quality reads. Then, clean reads were mapped to the reference genome of maize B73_RefGen_v4. DESeq was used to analyze differential expression genes (DEGs). *p* value < 0.05 and foldChange > 2 or foldChange < 0.5 were set as the threshold for significantly differential expression. The DEGs were blastx to the related species to establish a predicted protein interaction network. The Cytoscape v 3.7.1 app MCODE was used to develop the considerable modules in the network [50]. Advanced options were 2° cutoff, 0.2 Node Score Cutoff, and 5 K-Core.

### 4.3. Metabolite Extraction and LC-MS Analysis

An accurately weighed sample of 80 mg was transferred to a 1.5-mL Eppendorf tube. Two small steel balls were added to the tube; 20 μL of 2-chloro-l-phenylalanine (0.3 mg/mL) dissolved in methanol as internal standard and a 1-mL mixture of methanol and water (7/3, vol/vol) were added to each sample. Samples were placed at −80 °C for 2 min; then, grinded at 60 Hz for 2 min, and ultrasonicated at ambient temperature for 30 min after being vortexed, then placed at 4 °C for 10 min. Samples were centrifuged at 13000 rpm, 4 °C for 15 min; 300 μL of supernatant in a brown and glass vial was dried in a freeze concentration centrifugal dryer; 400 μL mixture of methanol and water (1/4, vol/vol) were added to each sample, samples were vortexed for 30 s, then placed at 4 °C for 2 min. Samples were centrifuged at 13000 rpm, 4 °C for 5 min. The supernatants (150 μL) from each tube were collected using crystal syringes, filtered through 0.22 μm microfilters, and transferred to LC vials. The vials were stored at −80°C until LC-MS analysis. QC samples were prepared by mixing aliquots of all samples to be a pooled sample.

An AB ExionLC UHPLC system (AB SCIEX, Framingham, MA, USA) coupled with an AB Triple TOF 6600 System (AB SCIEX, Framingham, MA, USA) were used to analyze the metabolic profiling in both ESI positive and ESI negative ion modes. An ACQUITY UPLC HSS T3 column (1.8 μm, 2.1 × 100 mm^2^) was employed in both positive and negative modes. The binary gradient elution system consisted of (A) water (containing 0.1% formic acid, *v/v*) and (B) acetonitrile (containing 0.1% formic acid, *v/v*) and separation was achieved using the following gradient: 0 min, 5% B; 2 min, 20% B; 4 min, 25% B; 9 min, 60% B; 14 min, 100% B; 16 min, 100% B; 16.1 min, 5% B and 18.1 min, 5% B. The flow rate was 0.4 mL/min and column temperature was 45 °C. All the samples were kept at 4 °C during the analysis. The injection volume was 5 μL. Data acquisition was performed in full scan mode (*m/z* ranges from 70 to 1000) combined with IDA mode. Parameters of mass spectrometry were as follows: Ion source temperature, 550 °C (+) and 550 °C (−); ion spray voltage, 5500 V (+) and 4500 V (−); curtain gas of 35 PSI; declustering potential, 80 V (+) and −80 V (−); collision energy, 10 eV (+) and −10 eV (−); and interface heater temperature, 550 °C (+) and 550 °C (−). For IDA analysis, the range of *m/z* was set as 25–1000, and the collision energy was 30 eV.

### 4.4. Data Processing and Differentially Accumulated Metabolite Identification

The acquired LC-MS raw data were analyzed by the progenesis QI software (Waters Corporation, Milford, MA, USA) using the following parameters. Precusor tolerance was set 5 ppm, fragment tolerance was set 10 ppm, and retention time (RT) tolerance was set 0.02 min. Internal standard detection parameters were deselected for peak RT alignment, isotopic peaks were excluded for analysis, and noise elimination level was set at 10.00, while minimum intensity was set to 15% of base peak intensity. The Excel file was obtained with three-dimension data sets including *m/z*, peak RT, and peak intensities, and RT–*m/z* pairs were used as the identifier for each ion. The resulting matrix was further reduced by removing any peaks with missing value (ion intensity = 0) in more than 50% of samples. The internal standard was used for data QC (reproducibility).

Metabolites were identified by progenesis QI (Waters Corporation, Milford, MA, USA) Data Processing Software, based on public databases such as http://www.hmdb.ca/, http://www.lipidmaps.org/ (accessed on 20 April 2021), and self-built databases. The positive and negative data were combined to get a combine data which was imported into the R tools package. Principle component analysis (PCA) and (orthogonal) partial least-squares-discriminant analysis (O)PLS-DA were carried out to visualize the metabolic alterations among experimental groups, after mean centering (Ctr) and Pareto variance (Par) scaling, respectively. The Hotelling’s T2 region, shown as an ellipse in score plots of the models, defines the 95% confidence interval of the modeled variation. Variable importance in the projection (VIP) ranks the overall contribution of each variable to the OPLS-DA model, and those variables with VIP > 1 are considered relevant for group discrimination. In this study, the default 7-round cross-validation was applied with 1/seventh of the samples being excluded from the mathematical model in each round, in order to guard against overfitting. The differential metabolites were selected on the basis of the combination of a statistically significant threshold of variable influence on projection (VIP) values obtained from the OPLS-DA model and *p* values from a two-tailed Student’s t test on the normalized peak areas, where metabolites with VIP values larger than 1.0 and *p* values less than 0.05 were considered as differential metabolites.

### 4.5. Gene Excavation and Location and RT-PCR Expression Analysis

Forty-eight genes were selected to perform qPCR according to reported regulating stomatal development key genes. Total RNA was isolated using the RNAqueous^®^ Total RNA Isolation Kit AM1912 (Life Technologies Corp., Grand Island, New York, NY, USA). The RNA yield was determined using a NanoDrop 2000 Spectrophotometer (Thermo Scientific, Waltham, MA, USA), and the integrity was evaluated using agarose gel electrophoresis with ethidium bromide stain. Quantified reactions were performed in a GeneAmp^®^ PCR System 9700 (Applied Biosystems, Waltham, MA, USA). RT-PCR was performed using LightCycler^®^ 480 II Real-time PCR Instrument (Roche, Basel, Switzerland) with QuantiFast^®^ SYBR^®^ Green qPCR Master Mix (Qiagen, Düsseldorf, Germany). Each sample was prepared in three copies for analysis. The primer sequences were designed in the laboratory and synthesized by Generay Biotech (Generay, Shanghai, China) based on the mRNA sequences obtained from the NCBI database. GAPDH was used as a reference gene. The sequences for primers used in qPCR are listed in Table S4. Genes were annotated by Nr, Nt, Pfam, KOG/COG, Swiss-Prot, KO, and GO databases, and then applied into the GO enrichment analysis and KEGG pathway analysis.

### 4.6. Drawing and Statistical Analysis

R toolkit was used to draw the pictures of transcriptome analysis and metabonomic analysis. Heatmaps were drawn with the Tbtools [51]. The analysis of phylogenetic tree was carried out by MAGA X [52] and depicted by EvolView online tools [53]. Signal networks were depicted by Adobe Illustrator CC 2019 (Adobe Systems Incorporated, San Jose, CA, USA). Interaction networks of genes, proteins, and metabolites were drawn by Cytoscape 3.5.1 [47]. ANOVA was used to analyze the significant differences between the measured data by comparing their means. The significance level was set at 0.05 (α). Excel 2016 (Microsoft Corporation, Redmond, Washington, DC, USA) was used for multiple comparisons to determine the least significant difference at 0.05 (α)

## 5. Conclusions

Research on the leaf stomatal development of monocotyledons is too complex to clarify. The influence of specific signals, such as blue light, on signal transduction is unpredictable and variable because of the complexity of the signal cascade. Using a combination of transcriptome and metabolomic analyses can help sort out the context in a complex network. In this study, we mapped a part of signal networks by transcription and metabolic analyses, and obtained eight genes that correspond to signal transduction and two related metabolite networks. The identification of these genes and networks will help complete the existing research on the stomatal development of maize.

## Data Availability

Not applicable.

## Supplementary Materials

The following are available online at https://www.mdpi.com/article/10.3390/ijms22105393/s1. Table S1: Summary of transcriptome analysis library, Table S2: The list of 53 genes classified by “signal transduction” and “environmental adaptation” in KEGG, Table S3: Classification list of KEGG level 2, enrichment term and annotation of 28 genes that interact with two classifications: “Signal transduction” and “Environment adaptation”, Table S4 Seq for primers in qPCR, Figure S1: The distribution map (A), correlation analysis (B) and principal component analysis (C) of genes expression, and cluster diagram of sequencing samples (D), Figure S2: PCA and heatmap of QC of metabolic.
