# Supplementary material for: Transcriptome and Metabolomic Analyses Reveal Regulatory Networks Controlling Maize Stomatal Development in Response to Blue Light
Source: Int J Mol Sci. 2021 May 20;22(10):5393. doi: 10.3390/ijms22105393 (PMC8161096; doi:10.3390/ijms22105393)
Supplement: Supplementary file 1 [file ijms-22-05393-s001.zip › Table S1 summary of transcriptome analysis library.pdf]

Sample	Raw reads	Raw bases	Clean reads	Clean bases	Valid bases	Q30	GC
Blue1	57.27 M	8.59 G	55.94 M	7.91 G	92.10 %	94.03 %	56.23 %
Blue2	53.26 M	7.99 G	52.03 M	7.35 G	91.96 %	94.05 %	56.81 %
Blue3	57.36 M	8.60 G	56.04 M	7.95 G	92.40 %	93.92 %	56.61 %
Red1	51.32 M	7.70 G	50.16 M	7.08 G	91.98 %	94.00 %	56.78 %
Red2	53.05 M	7.96 G	51.86 M	7.36 G	92.51 %	94.13 %	56.51 %
Red3	58.91 M	8.84 G	57.55 M	8.18 G	92.52 %	93.92 %	56.59 %
