# Supplementary material for: Transcriptome and Metabolomic Analyses Reveal Regulatory Networks Controlling Maize Stomatal Development in Response to Blue Light
Source: Int J Mol Sci. 2021 May 20;22(10):5393. doi: 10.3390/ijms22105393 (PMC8161096; doi:10.3390/ijms22105393)
Supplement: Supplementary file 1 [file ijms-22-05393-s001.zip › Table S2 list of KEGG classification of level 2 of the 55 DEGs.pdf]

NO.	Genes ID	type	KEGG classification	Enrichment term	Gene description or functional annotation
1	bZIP107	up	Signal transduction	Plant hormone signal transduction	Putative bZIP transcription factor superfamily protein; Transcription factor TGA4
2	gpm254	up	Environmental adaptation	Circadian rhythm - plant	Transcription factor HY5; Uncharacterized
3	gpm827	up	Signal transduction	Inositol phosphate metabolism	Putative phosphatidylinositol-4-phosphate 5-kinase family protein
				Phosphatidylinositol signaling system	
				Endocytosis	
4	gpm849	down	Environmental adaptation	Circadian rhythm - plant	Phosphatidylethanolamine-binding protein26; ZCN26
5	IDP491	down	Signal transduction	Plant-pathogen interaction	Respiratory burst oxidase homolog protein B
			Environmental adaptation	MAPK signaling pathway - plant	
6	LOC100192868	up	Environmental adaptation	Circadian rhythm - plant	LHY protein
7	LOC100217045	down	Signal transduction	Plant hormone signal transduction	Histidine-containing phosphotransfer protein 4
8	LOC100273422	up	Environmental adaptation	Plant-pathogen interaction	putative protein kinase superfamily protein
9	LOC100273598	up	Environmental adaptation	Circadian rhythm - plant	Two-component response regulator-like APRR9
10	LOC100274454	down	Signal transduction	MAPK signaling pathway - plant	ETHYLENE INSENSITIVE 3-like 5 protein
11	LOC100279342	up	Environmental adaptation	Circadian rhythm - plant	E3 ubiquitin-protein ligase COP1
				Ubiquitin mediated proteolysis	
12	LOC100279578	up	Signal transduction	Plant hormone signal transduction	PP2C11; 2C-type protein phosphatase protein
				MAPK signaling pathway - plant	
13	LOC100280135	up	Signal transduction	Amino sugar and nucleotide sugar metabolism	Basic endochitinase B
				MAPK signaling pathway - plant	
14	LOC100280474	down	Signal transduction	Plant hormone signal transduction	SAUR25 - auxin-responsive SAUR family member
15	LOC100281007	up	Signal transduction	Plant hormone signal transduction	SAUR14 - auxin-responsive SAUR family member
16	LOC100281015	up	Signal transduction	Phosphatidylinositol signaling system	caltractin
			Environmental adaptation	MAPK signaling pathway – plant	
				Plant-pathogen interaction	
17	LOC100281091	up	Environmental adaptation	Circadian rhythm - plant	Circadian clock associated1; Putative MYB DNA-binding domain superfamily protein

18	LOC100281845	up	Signal transduction	Plant hormone signal transduction	SAUR25 - auxin-responsive SAUR family member
19	LOC100281912	up	Signal transduction	Plant hormone signal transduction	Uncharacterized
20	LOC100283359	down	Signal transduction	Plant hormone signal transduction	two-component response regulator ARR3
21	LOC100283515	down	Environmental adaptation	Plant-pathogen interaction	caltractin
22	LOC100286122	up	Environmental adaptation	Ubiquitin mediated proteolysis	ubiquitin ligase protein COP1
				Circadian rhythm - plant	
23	LOC100381449	up	Signal transduction	Plant hormone signal transduction	SAUR-like auxin-responsive protein family
24	LOC103627330	up	Signal transduction	Plant hormone signal transduction	ETHYLENE INSENSITIVE 3-like 5 protein
				MAPK signaling pathway - plant	
25	LOC103627479	up	Signal transduction	Plant hormone signal transduction	SAUR11-auxin-responsive SAUR family member
26	LOC103630348	up	Signal transduction	Plant hormone signal transduction	gibberellin receptor GID1-like
27	LOC103631918	down	Signal transduction	MAPK signaling pathway – plant	auxin-responsive protein SAUR41
			Environmental adaptation	Plant hormone signal transduction	
				Plant-pathogen interaction	
28	LOC103632809	down	Signal transduction	Plant hormone signal transduction	auxin-responsive protein SAUR41
29	LOC103633012	up	Environmental adaptation	Plant-pathogen interaction	calcium-dependent protein kinase 22
30	LOC103633058	down	Signal transduction	Plant hormone signal transduction	transcription factor PHYTOCHROME INTERACTING FACTOR-LIKE 13
31	LOC103634871	up	Environmental adaptation	Circadian rhythm - plant	transcription factor HY5
32	LOC103636459	down	Signal transduction	Plant hormone signal transduction	transcription factor LG2-like
33	LOC103638645	up	Environmental adaptation	Circadian rhythm - plant	transcription factor HY5
34	LOC103641361	up	Signal transduction	Plant hormone signal transduction	transcription factor APG
			Environmental adaptation	Circadian rhythm – plant	
35	LOC103642058	down	Signal transduction	Plant hormone signal transduction	Auxin-responsive protein SAUR71
36	LOC103646211	up	Signal transduction	Plant hormone signal transduction	auxin-induced protein X10A
37	LOC103646787	up	Signal transduction	Plant hormone signal transduction	probable protein phosphatase 2C 37
				MAPK signaling pathway - plant	

38	LOC103648032	up	Signal transduction	Plant hormone signal transduction	probable indole-3-acetic acid-amido synthetase GH3.13
39	LOC103652527	up	Signal transduction	MAPK signaling pathway - plant	mitogen-activated protein kinase kinase kinase 18
40	LOC103654889	down	Signal transduction	Plant hormone signal transduction	Auxin-responsive protein SAUR71
41	LOC107326007	up	Signal transduction	MAPK signaling pathway - plant	Uncharacterized
42	LOC107403167	up	Signal transduction	MAPK signaling pathway - plant	Uncharacterized
43	LOC542189	up	Environmental adaptation	Plant-pathogen interaction	salt-inducible putative protein serine/threonine/tyrosine kinase
44	pco069505(751)	up	Signal transduction	Plant hormone signal transduction	snrkll1; Serine/threonine-protein kinase SRK2C
45	pco123854	up	Signal transduction	Plant hormone signal transduction	Auxin response factor
46	pco153543(105)	down	Environmental adaptation	Protein processing in endoplasmic reticulum	HSP90-2; HSP protein
				Plant-pathogen interaction	
47	PP2C14	up	Signal transduction	Plant hormone signal transduction	Uncharacterized
				MAPK signaling pathway - plant	
48	TIDP2950	up	Environmental adaptation	Plant-pathogen interaction	Putative calcium-binding protein CML25
49	umc1534	up	Signal transduction	Plant hormone signal transduction	Auxin-responsive protein
50	ZCN16	up	Environmental adaptation	Circadian rhythm - plant	ZCN16 protein
51	ZCN18	up	Environmental adaptation	Circadian rhythm - plant	ZCN18 protein
52	ZCN19	up	Environmental adaptation	Circadian rhythm - plant	ZCN19 protein
53	ZCN25	up	Environmental adaptation	Circadian rhythm - plant	ZCN25 protein
