# Supplementary material for: Transcriptome and Metabolomic Analyses Reveal Regulatory Networks Controlling Maize Stomatal Development in Response to Blue Light
Source: Int J Mol Sci. 2021 May 20;22(10):5393. doi: 10.3390/ijms22105393 (PMC8161096; doi:10.3390/ijms22105393)
Supplement: Supplementary file 1 [file ijms-22-05393-s001.zip › Table S3. List of KEGG classification of of 28 genes.pdf]

Gene ID	group	state	Gene description strived from NCBI
dhs2	B	up	deoxyhypusine synthase 2
IDP1436	B	up	4-coumarate--CoA ligase-like 9
LOC100281746	B	up	glycerol 3-phosphate permease
LOC100285616	B	down	calcineurin B-like protein 4
LOC103626304	B	up	protein ACTIVITY OF BC1 COMPLEX KINASE 7
LOC103646055	B	down	cytochrome P450 87A3-like
LOC103648196	B	down	putative glutaredoxin-C14
pco105718	B	up	Serine/threonine-protein phosphatase
PPCK1	B	up	phosphoenolpyruvate carboxylase kinase 1
umc1774	B	down	starch phosphorylase1
LOC100280217	C	up	uncharacterized
LOC103630810	C	up	RNA polymerase sigma factor sigE
LOC103636244	C	up	protein SPA1-RELATED 4
LOC100191429	D	up	putative MAP kinase family protein
LOC100279221	D	up	Protein phosphatase 2C and cyclic nucleotide-binding/kinase domain-containing protein
LOC100285849	D	up	uncharacterized
LOC103635843	D	up	CBL-interacting protein kinase 19
pco153236	D	up	Glycogen synthase kinase-3 MsK-3
TIDP9234	D	down	CBL-interacting kinase
LOC100286321	E	up	copper transporter 1
LOC100304064	E	down	SAUR33
LOC107548106	E	down	SAUR33
dhn1	F	up	dehydrin DHN1
LOC103641704	F	up	zeaxanthin epoxidase, chloroplastic
LOC103645618	F	up	carotenoid 9,10(9',10')-cleavage dioxygenase
LOC103649550	F	up	S-type anion channel SLAH3
LOC103651474	F	down	S-type anion channel SLAH2
wc1	F	up	white cap1
