# Supplementary material for: Transcriptome and Metabolomic Analyses Reveal Regulatory Networks Controlling Maize Stomatal Development in Response to Blue Light
Source: Int J Mol Sci. 2021 May 20;22(10):5393. doi: 10.3390/ijms22105393 (PMC8161096; doi:10.3390/ijms22105393)
Supplement: Supplementary file 1 [file ijms-22-05393-s001.zip › Table S4 seq for primers in qPCR.pdf]

**Gene Symbol**	**Forward primer (5' to 3')**	**Reverse primer (5' to 3')**
ZmCRY1	GTTCAACCTGCTATCCACG	AGTTCGGTCTCAGCTCTAA
ZmCRY2	TGAGAGTGACGCACTAGG	GTGCGGATCAAACTTGTACC
ZmPhyA1	ACTGCAAACAAGTGCCAA	GCATTTCCCATTCTCCGC
ZmPhyB1	GTACACAGGCGGCAAATG	CATGGACTCTGATCTTGAAACC
ZmPhyB2	ATGGTTGTCATTCACAATGCTA	CCTGCACATACTTTCCATTCTT
ZmSTOMAGEN	ATGCCTAGCTCAGGTTATGGA	GGCCATTCATACTTTCAGATGT
ZmEPF2	AGGTAGCACTACTTGAACCTT	TTCCGAACCACCGTGATT
ZmCOP1	CGCGTGTATGTCGTACTG	AATTCATGCCCTAACCGGA
ZmPIF4	TCTCCTTCCTTCCGAACG	ATGCAGCTAGATGACCCA
ZmHY5	CAAGAGCTAATGAGAATTGAGGGAC	CCAACAATGGTCAGGTGAGGC
ZmTMM	AACTCCGGTGGCGGTACTC	GCAAGGTGCAGACACACGAA
ZmER	GTCCCTACTGACAACAACTTC	ACCAAGCCAATATCCACAGA
ZmERL1	ACTGGAGATGTCTCTTCACT	TACCCAAGAAGCTGTCGG
ZmERL2	TTAGGCTTCGTCATATTGCTCT	GTCGGATGCTTTCTCAGGTA
ZmYDA	CCATTGAGCATCGCTCAC	GGACTTGATGGATTCTCTGTTC
ZmMKK4	ATGTTCGCCATCTGCTAC	CAGAGGCTGATGAAGTTCTTG
ZmMPK3	CTGTTCTGCTTGCTGCTGA	GCTGTGGCTGCCTTATTAT
ZmPAN1	GGTGCAATCCAACGCATAA	ACCGTCTCTGAAATCCACA
ZmPAN2	CTACCTAAACCTTTCCCGCA	TCAGATCAAGCACGGTAAGT
ZmPOLAR	CTACCGGGATGGAGTTGAT	GAGCCTTCTCTCCAACTGT
ZmSPCH	GCAGGCCAAATTCGTTACTATCTAC	GTGGCCTCTGCATCTTGAGATAA
ZmMUTE	AGTGTTGAGGATCTTGCCTACGA	CTCCTCCTGCGGCTTCTG
ZmFAMA	GCTGATCAAGACCATCGC	TTGAAGGAGTAGAGGACGG
ZmCDKB1-1	CGTCCCTGTAAAGAAGTACAC	CTGCACAAACAATTCAGACTC
ZmCYCD4-1	TCTGAATGCAGGGTGCTAGTA	CTGCCTTTAGTTTGGTCGT
ZmCyclin-A2	GAGGGAGTTCACTTGTGGA	TCTGCTTCTCTTTCTGTGCC
LOC107326007	CAGCGCGGCCAATAATAGA	ACACTTGCTGTAATTTATGCAC
LOC100192073	GTTCTTGGTTGGTTTGGTTTTCTG	GTGAACCGGGCTCCCATT
LOC100285849	CTGTTGGAGCCAGCCTTACAC	CGAAGCAACAACGGAAACTTTA
LOC100280217	TTCTTGACTCGCGGTGATATGT	TGCAGGCGACTTGACGATT
LOC100281912	TCCCGGCCGTAAAAATATGTA	TGAAGCAGCCATTTTACTGGTGTA
LOC107403167	ACATGATGGCATTTAGCAACTGA	ATGTACTCCACCGGCCATTG
LOC103641704	ACCAGTCACCATCCCATCATC	GGAGTATCGTGTATCTCCCGATTG
umc1774	AGGCGTACCGAGATCAGAAGTT	GATGAGCCAGCCGTGTTGA
ZmGAPDH	TATGTCCTTCCGTGTTCCTAC	AGCAGCCTTAATAGCTTTCTT
