# Supplementary figures and images for: Transcriptome and Metabolomic Analyses Reveal Regulatory Networks Controlling Maize Stomatal Development in Response to Blue Light
Source: Int J Mol Sci. 2021 May 20;22(10):5393. doi: 10.3390/ijms22105393 (PMC8161096; doi:10.3390/ijms22105393)

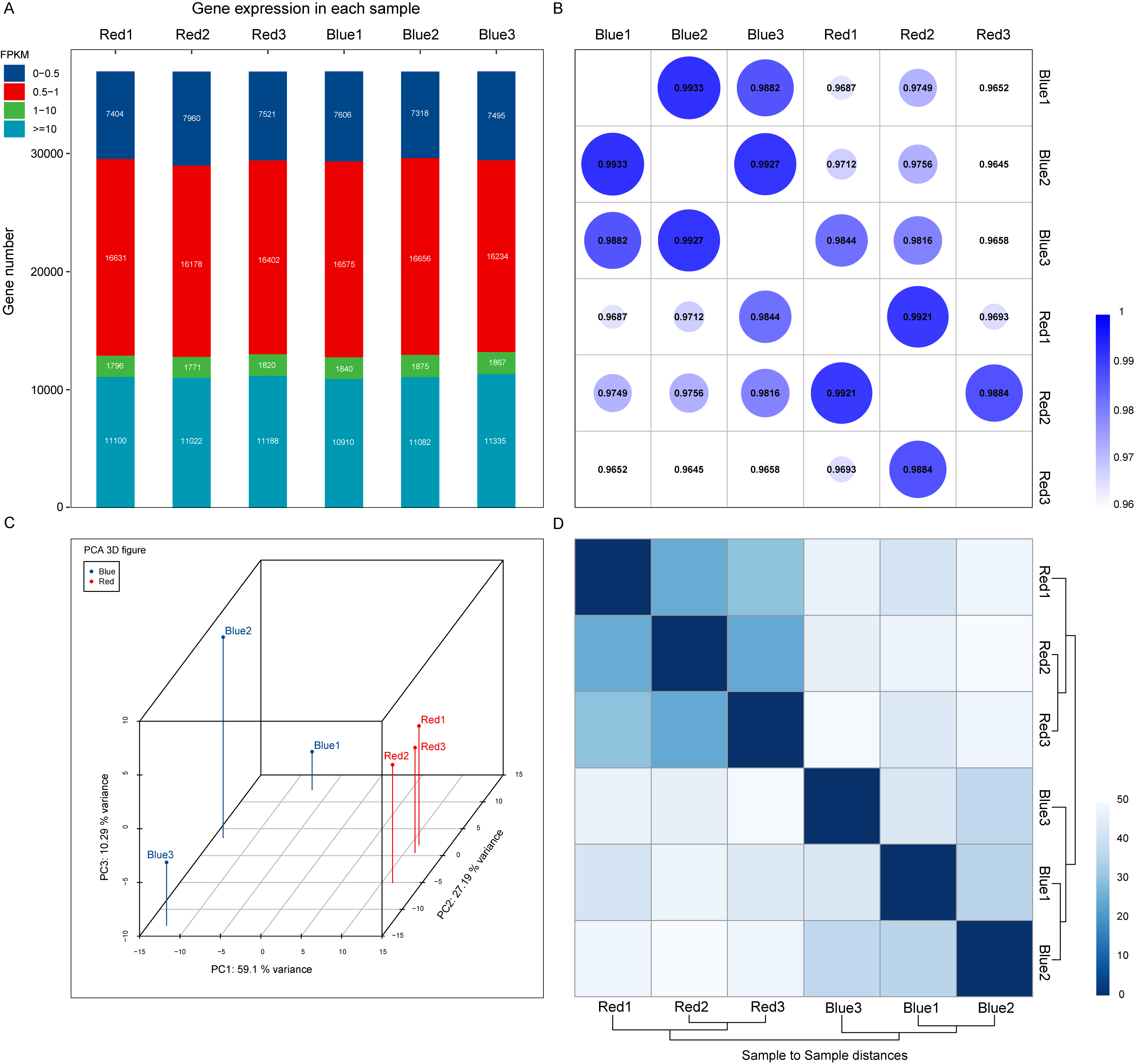

Supplement: Supplementary file 1 [file ijms-22-05393-s001.zip › Figure S1.tif]

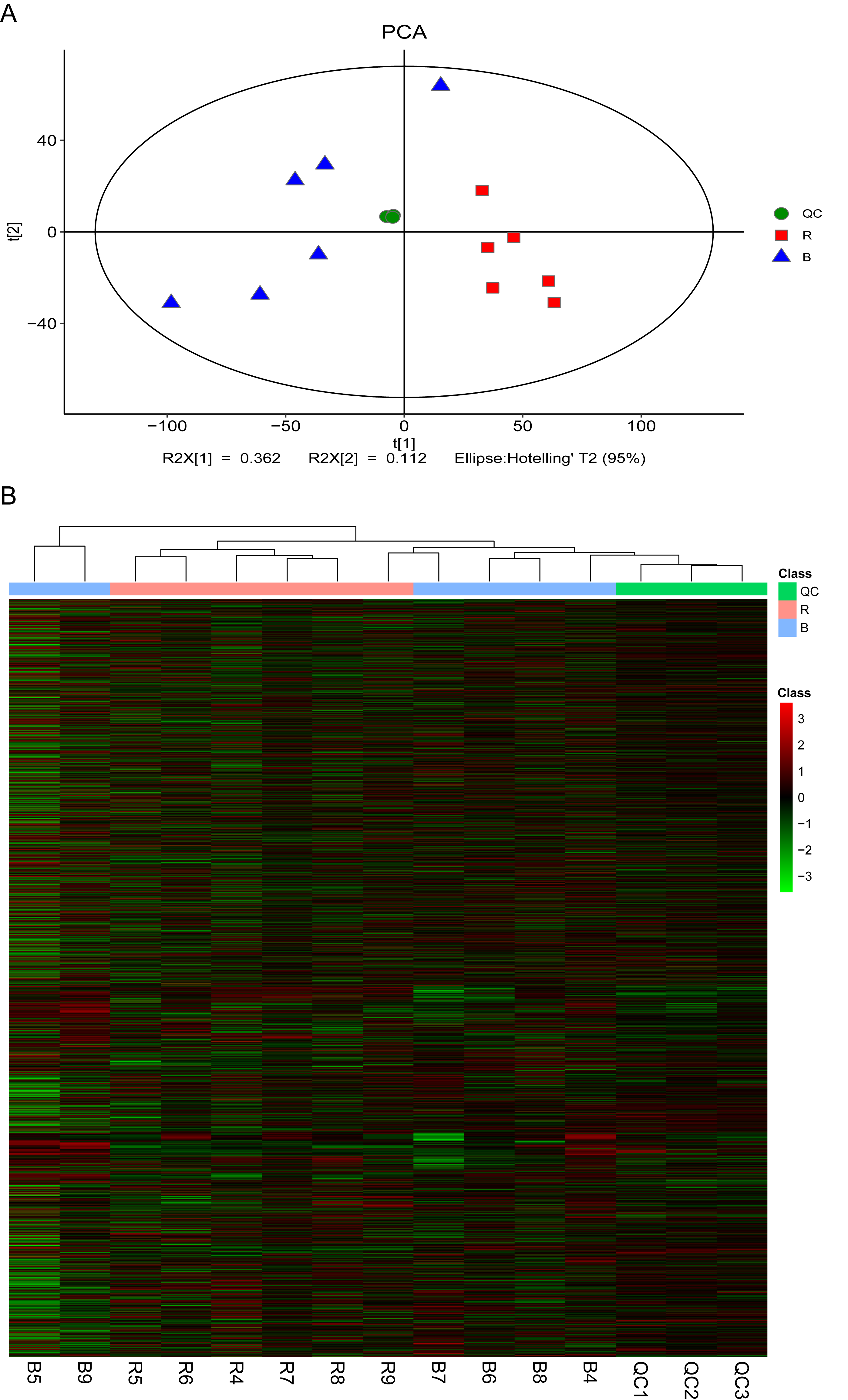

Supplement: Supplementary file 1 [file ijms-22-05393-s001.zip › Figure S2.tif]
